# Genetics and nutrition impacts on herd productivity in the Northern Australian beef cattle production cycle

**DOI:** 10.1016/j.vas.2021.100228

**Published:** 2021-12-26

**Authors:** Aduli E.O. Malau-Aduli, Jessica Curran, Holly Gall, Erica Henriksen, Alina O'Connor, Lydia Paine, Bailey Richardson, Hannake van Sliedregt, Lucy Smith

**Affiliations:** Animal Genetics and Nutrition, Veterinary Science Discipline, College of Public Health, Medical and Veterinary Sciences, Division of Tropical Health and Medicine, James Cook University, Townsville, Queensland 4811, Australia

**Keywords:** Northern Australia, Beef production, Genetics, Nutrition, Carcass quality

## Abstract

Genetics and nutrition drive herd productivity due to significant impacts on all components of the beef cattle production cycle. In northern Australia, the beef production system is largely extensive and relies heavily on tropical cattle grazing low quality, phosphorus-deficient pastures with seasonal variations in nutritive value. The existing feedlots are predominantly grain-based; providing high-energy rations, faster turn-off and finishing of backgrounded cattle to meet market specifications. This review focusses on the beef cattle production cycle components of maternal nutrition, foetal development, bull fertility, post-natal to weaning, backgrounding, feedlotting, rumen microbes and carcass quality as influenced by genetics and nutrition. This student-driven review identified the following knowledge gaps in the published literature on northern Australian beef cattle production cycle:

1. Long-term benefits and effects of maternal supplementation to alter foetal enzymes on the performance and productivity of beef cattle;

2. Exogenous fibrolytic enzymes to increase nutrient availability from the cell wall and better utilisation of fibrous and phosphorus deficient pasture feedbase during backgrounding;

3. Supplementation with novel encapsulated calcium butyrate and probiotics to stimulate the early development of rumen papillae and enhance early weaning of calves;

4. The use of single nucleotide polymorphisms as genetic markers for the early selection of tropical beef cattle for carcass and meat eating quality traits prior to feedlotting;

The review concludes by recommending future research in whole genome sequencing to target specific genes associated with meat quality characteristics in order to explore the development of breeds with superior genes more suited to the North Australian beef industry. Further research into diverse nutritional strategies of phosphorus supplementation and fortifying tropically adapted grasses with protein-rich legumes and forages for backgrounding and supplementing lot-fed beef cattle with omega-3 oil of plant origin will ensure sustainable production of beef with a healthy composition, tenderness, taste and eating quality.

## Highlights

• Northern Australia accounts for 49% of the national beef cattle herd.
• Harsh environmental conditions make nutrition challenging and impact herd productivity.
• Phosphorus deficiency contributes to low fertility rates and poor turn-off weights.
• Improvement of carcass quality in tropical beef cattle needs marker assisted selection.
• Knowledge gaps from the published literature to inform future research summarised.

## Introduction

Beef production in Northern Australia is reliant on an extensive grazing system in a geographical region dominated by prolonged droughts and highly variable rainfall (Mwangi et al., 2022). Thus, sustainable and profitable beef herd productivity is driven by strategies that adequately manage recovery, response and resilience to persistent drought. Bowen and Chudleigh (2021a) suggested key strategies for improving and sustaining extensive livestock production systems that are drought resilient and more profitable across northern Australia's beef enterprises. These include drought-preparedness (Rolfe et al., 2021), optimal steer sale age, breeder body condition and herd structure, effective phosphorus supplementation (Bowen et al., 2020) and improvement in steer nutrition through tropically adapted perennial legume grass pastures (Mwangi et al., 2021a; Mwangi et al., 2021b). Fordyce et al., (2021) defined the primary business measure of liveweight production for beef cows in northern Australia and reported that liveweight production in breeding beef cows was primarily governed by available nutrition and mating outcome. Bowen & Chudleigh (2021b) utilised a farm-management economics framework to conduct a contemporary assessment of the profitability and resilience of alternative livestock enterprises in the semiarid rangelands of northern Australia. They reported that meat sheep and rangeland meat goat enterprises produced the greatest rate of return on total capital where existing small ruminant enterprises in the semiarid rangelands of Queensland were profitable and resilient alternatives. Based on contemporary prices, they also found that meat sheep and rangeland meat goat enterprises produced the greatest rate of return on total capital. (Pearson, Filippi and Gonzalez, 2021) investigated the relationship between satellite-derived vegetation indices and liveweight changes of beef cattle in northern Australia. Their results suggested that the remote monitoring of pasture availability, liveweight and liveweight change of breeding herds under extensive grazing conditions could be used to increase productivity and land management in extensive beef production (Pearson, Filippi and Gonzalez, 2021). However, in order to specify management guidelines for sustainable grazing of regional land types retired from cultivation, Paton et al., (2021) suggested the need for assessing the productivity and sustainability of beef production from brigalow grazing lands, sown pasture growth and carrying capacity.

In 2021, the Organisation for Economic Co-operation and Development (OECD) projected that an 11% global population increase by 2030 will support a 14% increase in global meat consumption (OECD/FAO, 2021). A projected two million tonne increase in global beef consumption by 2024 has major implications for the Australian beef industry which contributes 4% of global beef production (MLA 2020)(MLA 2021). Genetic and nutritional improvements throughout the Australian beef production cycle will underpin the industry's ability to meet projected demand. As one of the major global meat producers, in 2020 Australia was the seventh highest beef producing country, and the second highest exporter of beef after Brazil (MLA, 2020), (MLA 2021). The beef cattle industry makes a significant contribution to the Australian economy, accounting for 23% of total gross farm production value in 2019–2020, and 23% of total export value (MLA, 2020). In 2019–2020 the gross value of the Australian beef cattle herd accounted for 23% of total farm value, at $15.1 billion (MLA, 2020). At present, the national beef cattle population is 23 million head, occupying approximately 50% of Australian farms, and 75% of agricultural land (MLA, 2020). The national beef cattle herd is largely distributed in northern Australia across Queensland, and Western Australia and the Northern Territory, which hold 43% and 16% respectively (MLA 2021). In 2017, the Queensland beef herd comprised 1.1% of the global beef population, and in 2018 it accounted for 8% of beef exports globally (DAFF 2021; MLA 2020,2021b). Cattle in northern Australia are bred for ectoparasite and heat resistance, and predominant breeds include Brahman, Droughtmaster, and Santa Gertrudis, which are well suited to the harsh environmental conditions but produce a different meat compared to breeds used in temperate operations (Greenwood, Gardner and Ferguson, 2018). There are over 10,000 beef production operations in Northern Australia, accounting for approximately 60% of the national cattle herd (Greenwood, Gardner and Ferguson, 2018). Regardless of the increased geographical size of Northern beef systems, their net production value is similar to their Southern counterparts, which utilise more productive sown pastures and increased market access. A significant increase in the Eastern Young Cattle Indicator from 428c/kg to 1,031c/kg since 2011 has been largely driven by climate factors and strong demand in the restocker market, fuelled by beef producers trying to rebuild their herds following drought, despite a significant proportion of Australia remaining drought declared (Australian Government - Department of Agriculture 2021; MLA 2021; Nason, 2021).

Australian beef production systems occupy over 200 million hectares (PricewaterhouseCoopers 2021), ranging from extensive pasture based systems in semi-arid and tropical rangelands, to intensive systems on irrigated pastures and feedlots (Yapp et al., 2001). Variation in annual rainfall characterises beef production in the Northern beef industry which relies on summer rainfall, in contrast to the Southern beef industry with year-round rainfall (Keywood, Emmerson and Hibberd, 2016). Despite differences in rainfall seasonality, both industries rely on climate as the single most important productivity factor (The Australian Beef Sustainability Framework, 2021). Challenges in the Northern beef industry stem from a limited ability to finish cattle and meet critical mating weight within the ideal timeframe, poor reproduction rates, poor feed conversion rates, variable water availability (DAFF 2021), and poor quality nutrition. Nutrition is affected by phosphorus and nitrogen deficient soils (Hopkins & Sullivan, 2021; Bowen et al., 2020) and unimproved native pastures (Chilcott et al., 2020). The Australian drought conditions are likely to intensify in frequency, length and portion of land covered, reducing land to an unproductive desert-like mass, which poses a challenge to future beef production (DAFF 2021).

Meat & Livestock Australia, an independent research and development body, has extensively funded research into genetic improvements in the beef cattle industry. Findings are regularly made available to producers to help them meet production targets, turn off a high quality product, and meet consumer demands in a range of markets (MLA 2021). Increased access to technologies allows beef producers to implement management strategies based on proven science, leading to increased sustainability and product consistency. The Estimated Breeding Values (EBV), a tool used by beef producers to make more informed decisions about sire selection by objectively predicting the heritable traits that will be passed onto progeny (Yapp et al., 2001), has been in use by the beef industry. As bulls contribute 50% to their progeny's genetics, it is important to select sires based on predicted genetic potential rather than phenotypic appearance only. The use of EBV in Australian beef production prioritises achieving high carcass weight and quality, based on seven estimated traits: carcass weight (kg), eye muscle area (sq. cm), rib fat (mm), rump fat (mm), retail beef yield (%), intramuscular fat (IMF) (%) and shear force (kg) (MLA 2021). In combination with artificial insemination, it allows producers to inseminate a large number of cows with the same bull's semen, which directly influences both the number of females served and the genetics used (MLA 2021). Crossbreeding with Bos taurus cattle in northern beef enterprises has been found to influence pre- and post-weaning growth, feedlot performance, and meat tenderness (FutureBeef, 2018).

The selection and management of genetically superior breeding stock that are well adapted to the environment whilst maintaining meat quality specifications, strongly influence productivity in the northern beef industry (FutureBeef, 2018). Nutritional strategies play a crucial role in animal performance and meat quality, and the inclusion of improved legumes in pasture-based grazing systems prior to feedlot finishing, has been found to improve the flavour, tenderness and juiciness of meat (Mwangi et al., 2019). Nutrition is a largely limiting factor in the northern beef industry, and the use of hormonal growth promotants (HGP) is a widely adopted practice which speeds up liveweight gain so that animals can be sold at a younger age (FutureBeef, 2019). However, the use of HGP is banned in the European Union and Tasmania, and treated cattle are noted to be of a variably reduced eating quality, based on Meat Standards Australia sensory testing (Business Queensland, 2021; MLA, 2019).

The primary objective of this paper is to review the published literature of the northern Australian beef cattle production cycle components of maternal nutrition, foetal development, bull fertility, post-natal weaning, backgrounding, feedlotting, rumen microbes and carcass quality as influenced by genetics and nutrition. The aim is to identify knowledge gaps for future research aimed at improving beef production.

## Maternal nutrition, foetal development & bulls

Northern Australian native pastures are characterized by poor nutritive value, low digestibility and high fibre content (Suybeng et al., 2019; Suybeng et al., 2020; Suybeng et al., 2021a; Suybeng et al., 2021b). McCosker et al., (2020) monitored the reproductive performance of commercial northern Australia beef breeding herds and found that substantial opportunities to increase reproductive performance exist, if the causes of variation are able to be identified and alleviated. Investigations of puberty, foetal development and associations with heifer- and steer-production traits (Johnston et al. 2009), reproductive performance (Allen et al., 2020; Burns et al., 2013), early mating fertility (Cullen et al., 2016) and prediction of mortality and conception rates of tropical beef cattle genotypes in northern Australia (Mayer, McKeon and Moore, 2012) all depend on maternal nutrition. (Roberts et al., 2016) reported that feeding improved pastures during the fifth and sixth months of gestation extended female progeny production. Their study also revealed that protein supplementation during late gestation produced heifers that were heavier from the onset of weaning to their calving period, and returned higher pregnancy rates than heifers from unsupplemented cows. (Funston, Larson and Vonnahme, 2010) demonstrated that supplementation during the last trimester improved heifer productivity by reducing pubertal age and increasing pregnancy rates. Pre-partum protein supplementation of first lactation heifers resulted in an increased re-conception rate (Schatz, 2015).

Rodríguez et al. (2021) administered inorganic copper to pregnant cows in late gestation resulting in an increase in progeny liveweight and average daily gain (ADG) until 75 days of age with no difference in finished steer carcass traits. Phosphorus supplementation compensates for increased bone reserve mobilisation in lactating cows, as production decreases if reserves are depleted (Dixon et al., 2020). Whilst supplementation aims to increase production, nutritional restriction could boost production when applied at the right stage of foetal development (Funston, Larson and Vonnahme, 2010).

Roberts et al. (2016) also found that supplementing cows in late gestation with less alfalfa produced progeny with increased body condition score that were 10 kg heavier at 5 years of age compared to their peers fed higher amounts of alfalfa. In contrast to Roberts et al. (2016), many other studies have reported that nutritional restriction had a detrimental effect on the placenta vasculature, architecture, organogenesis (Funston, Larson and Vonnahme, 2010; Long et al., 2021), foetal growth and birth weights (Funston, Larson and Vonnahme, 2010); (Vonnahme, Tanner and Hildago, 2018). (Vonnahme, Tanner and Hildago, 2018)found that the placenta and foetus within multiparous cows express an increased capacity to cope with nutritional restriction and stress than first-calf heifers. This is due to the function of altered uterine vasculature resulting from previous pregnancies enhancing the cow's ability to support future foetuses. The effect of maternal nutrition on reproductive traits of bull calves revealed a significant impact on total sperm defects (Polizel et al., 2021). This suggests that pre-partum supplementation strategies can be implemented to manipulate bull calf fertility and epigenetic factors that alter progeny gene expression.

Offering pre-pubertal bulls an enhanced plane of nutrition can lead to improved metabolic state that subsequently initiates an increase in the secretion of gonadotrophins and advances the onset of puberty (Kenny & Byrne, 2018). The beneficial effects of this unrestricted diet for 6 months or longer are not reversed by a moderate dietary restriction (Kenny & Byrne, 2018). It was found that feeding approximately 130% of maintenance energy and protein from 10 to 30 weeks of life led to an increase in testicular weight and production of sperm at 74 weeks, compared to bulls fed 100% of a maintenance energy and protein ration (Thundathil, Dance and Kastelic, 2016). Further research by (Thundathil, Dance and Kastelic, 2016) suggested that supplementing bulls during puberty does not compensate for nutrient restriction early in life, which is a key determinant of pubertal age, size of testis when sexual maturity is reached, and sperm production capacity.

Residual feed intake (RFI) is a moderately heritable trait that expresses the difference between actual feed consumed and predicted feed intake based on maintenance and growth; the lower the RFI value the more efficient the animal is ( Heida et al., 2021; Johnson et al., 2019; (Terry et al., 2021). Wang et al. (2012) reported that a large proportion of the bulls selected for RFI may fail to meet the minimum requirements for sperm motility. Johnson et al. (2019) found that bulls with a higher RFI had a larger scrotal circumference (SC) and corresponding sperm production as well as attaining puberty early when compared with low RFI bulls (Komatsu et al., 2015).

### Post-natal to weaning phase

It is essential for calves to receive 1.5 L of colostrum within the first 12 hours of life, because it contains immunoglobulins, fat, proteins, minerals and vitamins necessary for immune system development and vigour (Homerosky et al., 2017; Kessler, Bruckmaier and Gross, 2020). dos Santos et al., (2017) found that neonatal calves with poor vigour and an inability to suckle had subsequent poor weight gain and increased mortality risk. Increased suckling frequency in the first thirty days of life has been linked to higher ADG (Pires et al., 2021). Since the early 1990s (Miller et al. 1990; (Kerridge, Gilbert and Coates, 1990); McCaskill 1990; McLean et al. 1990; Kerridge 1990; (Wardsworth et al., 1990)) right through to 2020 (Bowen et al. 2020), low cost strategies for overcoming phosphorus deficiency in grazing systems based on both native and sown pastures in northern Australian beef production systems had been explored as a means of improving post-natal growth and average daily gains of beef cattle weaners. Wang et al. (2019) reported that conventional weaning based on age, typically from 180 to 210 days of age, decreases efficiency of production in the beef cattle industry (Wang et al., 2019). Weaning stress may have detrimental effects on the appetite of calves, ultimately affecting feed consumption, immune system and weight gain (Wang et al., 2019). Mattioli et al. (2020) investigated the effect of pre-weaning dam mineral supplementation including selenium, copper, zinc and Vitamins A and E. They demonstrated that supplementation prevented a decrease in the total antioxidant status of calves, improved antibody response and subsequent calf productivity at weaning (Mattioli et al., 2020).

A key factor in the feed intake and weight gain of calves is digestibility of solid feed (Wang et al., 2019). Calves weaned on the basis of age may not have complete rumen development and thus poor roughage digestibility (Wang et al., 2019). Wang et al. (2019) reported that calves consuming 750grams of solid feed per day had a greater antioxidant capacity, compared to those consuming 500grams. This increased antioxidant capacity can translate into enhanced ADG, dry matter intake, and feed efficiency. Improving the digestibility of pre-weaning calves through nutrient absorption by enhancing the development of rumen papillae has been investigated by Malau-Aduli et al., (2020) and Diao, Zhang and Fu, (2019). Strategies to improve rumen papillae development include but are not limited to, increasing available volatile fatty acids such as butyrate, altering the particle size of the diet and supplementation with probiotics (Malau-Aduli et al., 2020; Diao, Zhang and Fu, 2019; McCurdy et al., 2019).

Recent research into the effects of supplementing encapsulated calcium butyrate to pre-weaning calves (Malau-Aduli et al., 2020; McCurdy et al., 2019) evaluated ADG, chest girth, wither height, body length and body condition score and found that supplemented calves weighed on average, nine kilograms more and had an ADG that was 0.1 kg higher than the unsupplemented group (Malau-Aduli et al., 2020). The bodyweight (BW) of calves is moderately influenced by maternal genetic predisposition of the dam because the maternal heritability of BW is 0.42 in beef cattle (Cortés-Lacruz et al., 2017). Calves of low birth weight have been found to be significantly slower in postpartum growth compared to calves of high birth weight (Cortés-Lacruz et al., 2017; (Greenwood and Cafe, 2007)). Approximately half of the difference in ADG during feedlotting has been explained through calves of low birth weight versus a high birth weight (Greenwood and Cafe, 2007).

## Backgrounding

Backgrounding is a post-weaning feeding and growing program that produces cattle suitable to enter feedlots at approximately 280–400 kg (Mwangi et al. 2021a; Peel, 2003; Thomson & White, 2006). Backgrounding nutrition strategies support frame and muscle development as opposed to fattening (Mwangi et al. 2021b), and facilitate year-round supply of stocker cattle to feedlots (Gaughan et al., 2018; Groves, 2020). Poor management practices at calf processing and weaning may lead to increased disease incidence in feedlots (Groves, 2020). Bovine Respiratory Disease (BRD) negatively impacts carcass traits including ribeye area, marbling, tenderness, ADG, and final carcass weight (Vaughn et al., 2019; (Terry et al., 2021). Amat et al. (2020) found that treating calves with a single intranasal probiotic containing Lactobacillus strains increased their resistance and resilience to BRD pathogens (Holman et al., 2015). Limiting feed intake and restricting energy can prolong backgrounding, increases carcass weight potential by encouraging compensatory growth during finishing (Mwangi et al., 2019), but results in lower marbling (Johnson & DiCostanzo, 2017). However, if feed limitation causes weight loss, compensatory growth does not occur (Mwangi et al., 2019). Nutritional management during backgrounding influences ADG in stocker cattle, and programs metabolism and skeletal muscle characteristics and development in feeder cattle, impacting meat yield and quality (Vaughn et al., 2019).

Chibisa & Beauchemin (2018) found that the inclusion of early maturing corn silage up to 90% of dry matter intake (DMI) in backgrounding diets was a low cost feed alternative for barley silage with no loss to production and quality. Starch content of feed often exceeds small intestinal absorptive capacity, which has led to the formulation of diets that will increase ruminal starch fermentation, but increased dietary fermentable energy is linked to an increased risk of metabolic disorder development (Brake & Swanson, 2018). High-quality proteins like glutamic acid have been found to aid small intestinal starch digestion, and could be a beneficial inclusion in corn-based diets that would provide opportunity to increase intramuscular fat deposition (Brake & Swanson, 2018; Moriel, 2018). Van der Wal, Zeltwanger and DiCostanzo, (2019) found that including a molasses-based urea supplement in backgrounding diets increased DMI but had no effect on ADG, resulting in decreased FCE. Therefore, sugar supplementation may replace corn in pasture based backgrounding to increase DMI of long fibre roughage.

Feed conversion ratio (FCR) expresses the amount of feed required to produce 1 kg of liveweight (Heida et al., 2021; (Terry et al., 2021)). A feedlot study by (Fox, Tedeschi and Guiroy, 2001) found that a 10% FCR increase results in a 43% increase in profit, whilst a 10% ADG increase resulted in an 18% profit increase. A fibre utilisation study found that dietary inclusion of exogenous fibrolytic enzymes (EFE) increased bacterial attachment and stimulated microbial populations to enhance the digestion of cellulose, xylan and corn silage (Adesogan et al., 2019), but its effect is limited by enzyme degradation and individual variation (Arriola et al., 2017). Another trial included expansion-like proteins, which induced cell-wall relaxation, disrupted and weakened cellulose fibres, thus enhancing hydrolysis using cellulases and hemicellulases (Cosgrove, 2000; Georgelis, Nikolaidis and Cosgrove, 2014; Kim et al., 2012; Silveira & Skaf, 2016). The combined use of EFE and expansins increased cellulose and hemicellulose hydrolysis to 5 times more than if used singularly (Bunterngsook et al., 2015; (Kim et al., 2012); (Liu, Ma and Zhang, 2015)).

## Feedlots

### Dietary Protein

Cowley et al. (2019) proposed that by including minimum crude protein (CP) content and consequently overfeeding nitrogen, a low-cost and low-risk finishing diet would be produced. In North America, wet-corn distillers grain by-products were included as a cheaper source of protein and energy in finishing diets to replace cottonseed and soybean meal. The low rumen degradable protein (RDP) content of corn was subsidised by non-protein nitrogen (NPN) supplementation generating a major surplus of protein. Reports show that Australian finishing diets involve wheat or barley base with protein supplied by cottonseed, or less commonly by canola meal and lupins (Cowley et al., 2019). Dry Distillers Grains (DDG) are high in NDF and low in lignin content, thus providing readily digestible fibre, however, vary in other nutritional composition. Although good sources of protein, calcium, phosphorus and sulphur, DGGs require supplementation due to containing some undesirable components and a minimal soluble carbohydrate fraction (Cowley et al., 2019). A summary of the different nutritional properties of different grain types is presented in Table 1. Corn was the most commonly used in North America.

**Table 1:** Variation in nutritional characteristics of different grain-type sources of Dry Distillers Grains.

Source	Characteristics
Corn	Highest rumen undegradable protein; net energy
Wheat	Lowest rumen undegradable protein; highest crude protein
Barley	Lowest crude protein; highest neutral and acid detergent fibres
Sorghum	Highest ether extract, rumen undegradable protein
	

High protein DDGs (>34% CP) are produced by the removal of fibre or reducing non-fermentable components. This enhances amino acid digestibility. However, CP content has a negative correlation with phosphorus content, necessitating inorganic phosphorus supplementation (Buenavista, Siliveru and Zheng, 2021).

Low-oil DDGs have reduced digestible and metabolisable energy (ME) and amino acid digestibility, but a higher protein and fibre content. Up to 200 g of low-oil DGGs has been used to replace every kg of barley grain without detrimental effects on carcass quality and growth performance (Buenavista et al., 2021). Replacing soybean meal with 300 g/kg of de-oiled wet DGs improved overall feedlot performance including DM and CP intakes. Additional positive effects of decreasing the acetate: propionate ratio, maintaining the ruminal pH and preventing sub-acute ruminal acidosis (SARA) were observed by Carvalho et al., (2021). They further showed that the acetate:propionate ratio could also be reduced by supplementing a mix of extracts of baccharis, tamarind, cashew nut shell liquid which also enhanced DM, NDF and OM digestibility.

Slow-release urea (SRU) can be used to replace a portion of plant RDP by providing NPN (Salami et al., 2020; Salami et al., 2021). Salami et al. (2021) showed that SRU was superior to urea in producing microbial crude protein (MCP) and reduced the risk of ammonia toxicity. Although SRU had no effect on overall DM or CP intake, the positive effects on LWG and FE reduced the days on feed to slaughter and feed associated costs by 6% by reducing the inclusion rate of vegetable protein sources (Salami et al., 2020; Salami et al., 2021).

Growth promoters increase deposition of lean muscle mass, protein accretion and hormone mediated cellular proliferation alongside reducing protein degradation. Cumulatively, this increases dietary requirements for MP during compensatory growth in the early finishing period (Cowley et al., 2019). Beta agonists are thought to increase the requirements of CP by modifying protein metabolism on a cellular level as well as NPN requirements by upregulating protein synthesis (Cowley et al., 2019).

### Microbes

The rumen microbiome can cause up to 20% variation in production (Paz et al., 2018). A study found that approximately 34% of microbial taxa is relatively heritable (h^2^ ≥ 0.15) (Li et al., 2019). These traits were found to be associated with FCR, ADG and DMI (Li et al., 2019). Probiotics such as Yeast compete with lactate-producing bacteria for available sugars supplied by concentrate rich diets, supporting the growth of lactate-utilising bacteria and subsequently increasing the rumen pH. Through this mechanism, yeast has been used to treat cattle with SARA (Amin & Mao, 2021). Additionally, the inclusion of yeast in diets had a positive correlation with DMI, weight gain and FE and promoted the growth of cellulolytic bacteria that breakdown fibre rich feeds (Amin & Mao, 2021).

### Trace Minerals

The inclusion of trace minerals like zinc in association with chromium and methionine has been shown to enhance feed utilisation efficiency and positive effects on meat characteristics (Lee et al., 2020; Vellini et al., 2020). Vellini et al. (2020) suggested that supplementing cattle with amino acid chelated minerals resulted in increased ruminal amylase and cellulase activity while reducing methane production. Additionally, Lee et al. (2020) found that rumen boluses and dietary supplementation of 1–5 times the required amount of copper, cobalt, selenium and iodine resulted in greater DMI and ADG than in unsupplemented animals. Vitamin B12 is synthesised from Co and is involved in gluconeogenesis via propionate metabolism, thus decreased Co supply increases the propionate half-life which may decrease the DMI (Lee et al., 2020). A study by (Akanno et al., 2019) examined the sorting of animals on feedlot arrival according to their molecular breeding values and reported significant influences on lean meat yield and marbling. Allowing targeted feeding resulted in a more consistent final carcass product across each animal (Akanno et al., 2019).

## Carcass Quality

Meat quality is influenced by nutrition and genetics, which can be manipulated to achieve desirable eating quality traits including tenderness, flavour and juiciness, meat yield, and marbling score (Felderhoff et al., 2020); Malau-Aduli & Holman 2015a; Malau-Aduli & Holman 2014; Malau-Aduli et al. 2000a; Malau-Aduli et al. 2000b; Malau-Aduli et al. 2000c; Malau-Aduli et al. 2000d). Cooke et al. (2020) reported significant genetic correlations between muscle lipid content and marbling score, in which Bos taurus cattle had greater intramuscular and subcutaneous fat than Bos indicus cattle, and B. indicus demonstrated a reduced capacity to synthesise lipids resulting in lower marbling scores (Cooke et al., 2020). A comparison of meat quality in Limousine, Charolais, and Angus bulls showed that Limousine bulls had the highest protein and lowest fat contents, Charolais rated the best in colour, taste, and aroma, and Angus meat was rated the best for consistency (Sakowski et al., 2001; Malau-Aduli et al. 1997; Malau-Aduli et al. 1998). There is an emphasis on pH as it is a major determining factor of carcass quality, with the desired pH range of 5.4–5.8 (Muižniece & Kairiša, 2020). Heifers were found to have a more desirable meat pH (86 % of carcasses) when compared to bulls (65% of carcasses), however, there were no differences between pH across different age groups (Muižniece & Kairiša, 2020).

SCD, FASN, FABP4 and SREBPs are four genes which have become strong candidates for influencing the fat deposition and carcass composition in Eastern Asian cattle breeds and Australian temperate breeds ( Lee & Park, 2016; Komatsu et al. 2012; Raza et al., 2018; Komatsu & Malau-Aduli 2014; Malau-Aduli et al. 2015). Selection for enzymes such as fatty acid synthase (FASN) is reported to increase fat deposition in adipose tissue (Raza et a. 2018; Lee & Park 2016; Malau-Aduli et al. 2016; Malau-Aduli & Kashani 2015; Malau-Aduli & Holman 2015a; Malau-Aduli & Holman 2015b). Selection of these genes in northern Australian composite breeds could potentially improve intramuscular fat and eye muscle area thus improving meat quality.

Supplementation with organic sources of selenium, zinc, copper, and manganese resulted in an improvement in growth performance, health status, carcass quality, and overall meat quality due to higher levels of antioxidants in the meat (Rossi et al., 2020). Corn finished beef has been shown to contain greater amounts of oleic and total monounsaturated fatty acids, but lesser polyunsaturated fatty acids and omega-3 (Lafreniere et al., 2021; Malau-Aduli et al. 1997; Malau-Aduli et al., 1998). Essential oils were evaluated as an alternative to monensin beef cattle diets and their effect on feed intake, performance, carcass characteristics and ruminal fermentative parameters (Torres et al., 2021). They were found to be ineffective in protecting the liver against abscesses, but carcass dressing percentage, ribeye area, and subcutaneous fat thickness values increased (Torres et al., 2021). Arginine and lysine supplementation can have positive effects on serum amino acids, growth performance and carcass traits in which feedlot steers (Teixeira et al., 2019) and lot-fed sheep (Malau-Aduli et al. 2019) developed more muscle and leanness from lysine, whereas arginine only contributed to increased moisture content of steaks and decreased serum glutamate as well as decreased lysine after 87 days of feeding (Teixeira et al., 2019).

## Conclusion: Knowledge Gaps

From the published literature, it is evident that there is a relationship between foetal pancreatic and small intestine enzyme activities. Novel and innovative research is needed on the long term effects of maternal supplementation and alteration of foetal enzymes on post-natal growth performance and tropical beef cattle productivity. Similarly, the role of phosphorus supplementation in nutritional bovine physiology to reduce production losses in phosphorus-deficient pasture areas of tropical northern Australia needs further investigation. Exogenous fibrolytic enzymes and expansion-like proteins are currently used as short-term treatments to increase fibre utilisation of poor quality roughages. Further research is needed to establish its long-term effects on northern Australian beef cattle production, especially during prolonged droughts. Early calf weaning for faster and subsequently heavier turn-off weights through enhanced rumen papillae development at the pre-weaning stage can be targeted through supplementation with probiotics. This could potentially increase available butyrate, thus promoting rumen development, and in turn, enhance growth and productivity (Malau-Aduli et al., 2020). Yeast is another probiotic that requires further research into maximising the benefits of productivity when incorporated into ruminant diets. Dry distiller's grains represent a readily digestible source of fibre for feedlot rations, however, further investigation is required to maximise efficacy. Whole genome sequencing to target specific genes associated with meat quality characteristics (Pewan et al. 2021a; (Pewan et al., 2021b) (Pewan et al., 2020a); (Pewan et al., 2020b) are needed to further develop tropical cattle breeds with superior genes more suited to the North Australian beef industry (Mwangi et al. 2019). Specifically, the effects of FABP4, FASN, SCD and SREBP genes on intramuscular fat and carcass quality (Takahashi et al. 2016; Malau-Aduli et al. 2015; Kashani et al. 2015a; Kashani et al. 2015b) in northern Australian composite breeds needs to be researched further.

## Declaration of competing interest

None.
